# An Emerging Paradigm for Safer and Faster Recovery: A Narrative Review on Opioid Sparing Anesthesia in Surgery

**DOI:** 10.7759/cureus.95726

**Published:** 2025-10-30

**Authors:** Elmoatazbellah Nasr, Nervana Khalil, Maan Sarsam, Mohamed Omran, Ahmed Elhantiry

**Affiliations:** 1 General Surgery, Calderdale and Huddersfield National Health Service (NHS) Foundation Trust, Huddersfield, GBR; 2 Pediatric Surgery Department, Mansoura University Children Hospital, Mansoura, EGY; 3 General Surgery, Weston General Hospital, University Hospitals Bristol and Weston National Health Service (NHS) Foundation Trust (UHBW), Weston-super-Mare, GBR; 4 Anesthesia and Critical Care, The Memorial Souad Kafafi University Hospital, 6th of October City, EGY

**Keywords:** eras, ofa, opioid-free anesthesia, recovery, surgery

## Abstract

Opioid-free anesthesia (OFA) replaces opioid use with many non-opioid drugs, such as dexmedetomidine, lidocaine, esketamine, regional techniques, and enhanced recovery after surgery (ERAS)-aligned strategies to control pain while minimizing opioid-related adverse effects. Across surgical procedures, we conducted a narrative review of the literature, which showed that OFA is consistently associated with lower postoperative nausea and vomiting, faster recovery of gastrointestinal function, and reduced rescue opioid use, with similar post-anesthesia care unit (PACU) stay and pain scores in many trials. Pediatric and ambulatory settings also show fewer emetogenic symptoms and quicker readiness for discharge. However, evidence quality is mixed: several randomized trials and meta-analyses report meaningful reductions in PONV and opioid consumption but only modest or clinically marginal analgesic gains. Safety signals-particularly with α2-agonists like dexmedetomidine-include intraoperative hypotension/bradycardia and potential prolonged sedation, underscoring the need for careful dosing and patient selection. Contemporary guidance therefore favors opioid-sparing (minimizing rather than eliminating opioids) as a pragmatic interim goal while high-quality trials further define OFA’s net benefit, optimal drug combinations, and perioperative extensions (e.g., postoperative low-dose infusions). Future work should refine protocols that de-emphasize routine lidocaine, titrate dexmedetomidine judiciously, and integrate targeted regional blocks to balance recovery benefits with hemodynamic safety, particularly in high-risk populations such as those with obesity or sleep apnea.

## Introduction and background

Opioid-free anesthesia (OFA) is an intraoperative strategy that avoids opioid administration by using non-opioid drugs and regional techniques to control pain and surgical stress, aiming to reduce nausea, sedation, respiratory depression, and ileus that may occur with opioid use [1,2]. Opioids have long been a standard component of general anesthesia for pain management. However, increasing evidence suggests that opioid use during surgery can lead to postoperative hyperalgesia, which may result in chronic postsurgical pain (CPSP) and prolonged opioid dependence [3,4]. While classically used for pain relief, opioids may lead to serious problems such as respiratory depression, compromised GIT function, postoperative nausea and vomiting (PONV), itching, difficulty urinating, postoperative delirium, and dependence on opioids [3,5,6]. 

We are now gaining more interest in pain management and pain reduction perioperatively, aiming to provide effective pain relief while minimizing side effects. Opioid-free anesthesia (OFA) is a contemporary approach that utilizes a combination of non-opioid medications and techniques to achieve high-quality anesthesia without the use of opioids [7]. OFA is a technique that utilizes non-opioid medications and methods to achieve anesthesia while minimizing the use of opioids, which are associated with various risks. This approach aims to expedite recovery and reduce the duration of hospital stays. OFA is increasingly used in various surgical procedures and has shown effectiveness in pain management during and after surgery [8-13].

Non-opioid medications have proven effective in reducing systemic inflammatory response. For example, giving dexmedetomidine during some lung cancer surgeries has been shown to lessen surgical inflammation, oxidative stress, and pain after the operation, aiding recovery without increasing the chance of side effects or complications [14]. OFA aligns seamlessly with the principles of enhanced recovery after surgery (ERAS) as it may improve patient outcomes, decrease the occurrence of postoperative complications, and accelerate patient recovery [15].

## Review

Commonly used drugs

Dexmedetomidine, a highly selective α2-adrenergic agonist, offers a multifaceted approach to patient care. Its primary actions include providing effective sedation, reducing anxiety (anxiolysis), offering pain relief (analgesia), suppressing sympathetic nervous system activity (sympatholysis), and its related effects, significantly contributing to opioid-sparing strategies in various medical settings [16,17]. It yields sedation with minimal respiratory depression [18,19]. Perioperatively, it reduces opioid use, improves pain, lessens adverse events, and accelerates recovery [14,20-22]. As a local anesthetic adjuvant, it prolongs the block and reduces opioid requirements. In ultrasound-guided ulnar blocks, it resulted in a faster onset and longer duration of action compared to intravenous administration [23-25]. 

A systematic review and meta-analysis asked whether perioperative systemic α2-agonists (clonidine, dexmedetomidine) reduce postoperative opioid use and pain, and how they affect adverse events, nausea, recovery, and longer-term pain outcomes. Thirty randomized trials (1,792 adults) showed a 24-hour morphine-sparing effect: clonidine −4.1 mg (95% CI −6.0 to −2.2) and dexmedetomidine −14.5 mg (−22.1 to −6.8). Pain intensity at 24 hours was modestly lower (VAS 0-10 cm): clonidine −0.7 cm (−1.2 to −0.1), dexmedetomidine −0.6 cm (−0.9 to −0.2). Early postoperative nausea decreased (number needed to treat ≈9), and recovery times were not prolonged. Adverse effects differed: clonidine increased intra- and postoperative hypotension (NNH ≈9 and 20); dexmedetomidine increased postoperative bradycardia (NNH ≈3). No trials reported effects on chronic pain or hyperalgesia [26].

Lidocaine offers more than just local anesthetic effects; it also has strong pain-relieving, anti-inflammatory, anti-hyperalgesic, and gut motility-boosting properties [27-29]. Clinically, IV lidocaine helps reduce pain and the need for opioid administration for pain killing after surgery, which in turn lowers the chance of opioid side effects like nausea, vomiting, and constipation. In opioid-free anesthesia (OFA), lidocaine is often combined with other non-opioid medications like dexmedetomidine and esketamine [27-29]. Esketamine is an N-methyl-D-aspartate (NMDA) receptor antagonist that offers many benefits when used. It is effective for inducing general anesthesia in brief surgical procedures, providing pain relief, cardiovascular stability, and quick recovery [30,31]. Esketamine is frequently used in conjunction with other non-opioid medications like dexmedetomidine and lidocaine to create a multimodal anesthetic approach. This combination improves pain management after surgery and lowers the occurrence of opioid-related side effects such as nausea, vomiting, and respiratory depression [8,32].

Nonsteroidal anti-inflammatory drugs (NSAIDs) and clonidine are highly effective pain killers after surgery, significantly reducing the need for opioids. They offer several advantages in perioperative care, including decreased pain, nausea, and fatigue, and should be included in opioid-free anesthesia protocols [33]. Epidural anesthesia effectively manages pain during and after various surgeries, such as those involving the chest, abdomen, and bones. However, its popularity has decreased due to potential serious complications like catheter damage, unintended injection into the spinal fluid, infections, and blood clots around the spinal cord [34]. 

**Figure 1:**
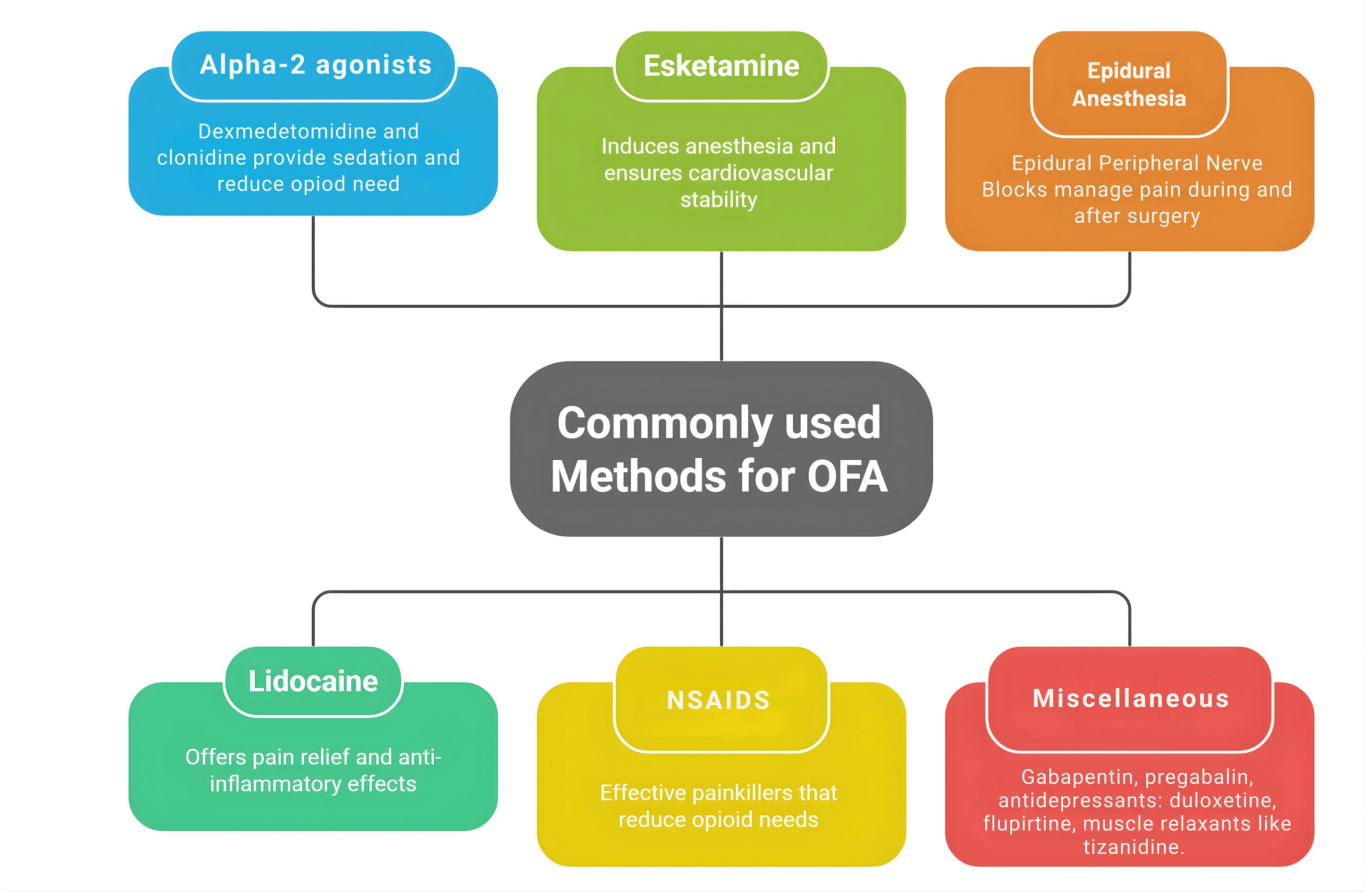
Commonly used drugs for OFA A figure created by the article authors summarising commonly used drugs for opioid-free anesthesia (OFA) according to references [16–41].

With ultrasound guidance becoming routine in anesthesia, peripheral nerve blocks are now widely used with improved safety through real-time visualization. By depositing local anesthetic next to specific nerves or plexuses to interrupt conduction, these blocks provide targeted analgesia. Evidence consistently shows they lower perioperative opioid requirements and enhance patient outcomes (Figure 1) [35-37]. There are many other medications that, when used perioperatively, provide opioid-sparing analgesia. They are anticonvulsants (gabapentin, pregabalin), antidepressants (duloxetine, fluoxetine), and muscle relaxants (tizanidine) [38-41].

Value for the reduction of perioperative adverse effects

Effective multimodal analgesia and anesthetic strategies are crucial for the adoption of ERAS protocols. These regimens are specifically developed to minimize postoperative pain and opioid use, both during and after surgery, which in turn reduces opioid-related side effects and facilitates a better recovery [42]. A previous study investigating a strict OFA protocol for cardiac surgery utilizing lidocaine, dexamethasone, and ketamine while omitting intraoperative opioids demonstrated significant postoperative benefits. The regimen successfully reduced 48-hour morphine consumption without increasing pain scores. Furthermore, this OFA approach was associated with accelerated recovery milestones, including faster extubation and shorter ICU stays [15]. These outcomes of opioid minimization and enhanced recovery directly align with the core objectives of ERAS. This strong alignment indicates that OFA is compatible with ERAS principles and can serve as an effective component within ERAS pathways, rather than being considered a separate alternative.

OFA avoids the μ-opioid-mediated suppression of enteric peristalsis, thereby supporting earlier restoration of gastrointestinal function. By relying on multimodal agents and regional techniques (e.g., dexmedetomidine, ketamine, lidocaine infusions, peripheral or neuraxial blocks), OFA reduces ileus risk, enables earlier oral intake, and accelerates bowel recovery [8,13,32]. Across a range of surgical procedures, OFA protocols are consistently linked to lower rates of PONV and more rapid GI recovery, aligning well with enhanced recovery pathways [13,32,43]. OFA has demonstrated benefits in pediatric patients undergoing procedures such as hand surgery and tonsillectomy. Studies have shown that children in the OFA group experienced a reduced incidence of PONV, decreased need for postoperative analgesics, and quicker achievement of discharge criteria compared to those in opioid groups [44-46].

Prospective trials have demonstrated a clear reduction in the prevalence of this complication among patients undergoing laparoscopic bariatric surgery. The primary motivations for the growing interest in OFA within the field of bariatric anesthesia are centered on enhancing patient safety and facilitating a more rapid and complete recovery. Specifically, OFA aims to circumvent the risks associated with opioid-induced respiratory depression (OIRD) and mitigate the occurrence of oversedation during the crucial postoperative period. By avoiding these common opioid-related complications, OFA seeks to improve patient outcomes significantly [47-49]. 

Opioid-sparing techniques have been identified as a potential method to reduce the risk of opioid-induced respiratory depression (OIRD), according to a meta-analysis focused on the contributing factors of OIRD [50]. The Enhanced Recovery After Bariatric Surgery (ERABS) recommendations advocate making multimodal, opioid-sparing analgesia the default approach. In practice, this means combining non-opioid medications-such as acetaminophen and NSAIDs, with adjuncts like gabapentinoids or low-dose ketamine when appropriate-with targeted regional anesthesia techniques (e.g., transversus abdominis plane or rectus sheath blocks). Using co-analgesics and regional methods in concert aims to deliver effective pain control while minimizing opioid requirements [51].

Hao et al. conducted a randomized trial of 80 adults undergoing laparoscopic cholecystectomy, OFA with opioid-based anesthesia. OFA yielded higher QoR-15 scores on postoperative days 1 and 2, fewer opioid-related symptoms, and differences in blood pressure with a hypotension signal. PACU stay, extubation time, bradycardia, and heart rate were similar. Overall, OFA improved short-term recovery versus OA [52].

A systematic review and meta-analysis conducted by Olausson et al., encompassing 1934 patients across 26 randomized controlled trials, investigated the efficacy of strict opioid-free general anesthesia versus opioid-based approaches. The study included various surgical procedures, such as laparoscopic gynecological, upper gastrointestinal, and breast surgery. Key findings revealed that opioid-free anesthesia significantly reduced adverse postoperative events (odds ratio (OR) 0.32, 95% CI 0.22 to 0.46, I2 = 56%, p < 0.00001). This reduction was primarily driven by a decreased incidence of postoperative nausea (OR 0.27, 0.17 to 0.42, p < 0.00001) and vomiting (OR 0.22, 0.11 to 0.41, p < 0.00001). Furthermore, postoperative opioid consumption was significantly lower in the opioid-free group (−6.00 mg, −8.52 to −3.48, p < 0.00001). Notably, the study found no significant differences between the two groups regarding the length of stay in the post-anesthesia care unit or overall postoperative pain levels [53].

Da Silveira et al. conducted a systematic review and meta-analysis of randomized trials testing opioid-free anesthesia (OFA) for laparoscopic abdominal surgery, defining OFA as no opioid use preoperatively or intraoperatively. The prespecified primary outcomes were postoperative nausea/vomiting (PONV; nausea or vomiting) and intraoperative bradycardia; secondary outcomes included pain scores, rescue opioid use, and hospital stay. Across 26 RCTs (2,025 patients)-mostly cholecystectomy (9 trials) and bariatric surgery (8 trials)-OFA significantly lowered PONV risk (RR 0.55, 95% CI 0.40-0.74; P<0.001) without increasing bradycardia. Although pain scores and opioid consumption were statistically lower, the magnitude of these reductions was not clinically meaningful. Trial sequential analyses supported the PONV and bradycardia findings [54,55]

Guinot et al. performed a retrospective matched case-control study in cardiac surgery with cardiopulmonary bypass, comparing opioid-free anesthesia (lidocaine, dexamethasone, and ketamine) to sufentanil-based anesthesia. OFA halved 48-hour postoperative morphine use (median 5 vs 15 mg) with similar pain scores, reduced a composite of major adverse events, and shortened time to extubation and ICU stay, warranting prospective confirmation in randomized trials [15].

Bowel Function

Research indicates that elderly patients receiving dexmedetomidine during abdominal surgery experience a notable reduction in the time until their first flatus and bowel movement, as well as a shorter overall hospital stay [23]. Sun et al. conducted a randomized controlled trial of 62 adults undergoing laparoscopic colorectal cancer resection, testing continuous subanesthetic esketamine versus control. Esketamine reduced postoperative fatigue (ICFS) on days 3 and 7 and improved mood (higher PANAS positive on day 3; lower negative on days 3 and 7). Handgrip strength, numeric rating scale (NRS) pain, Athens insomnia scale (AIS) sleep, neutrophil-to-lymphocyte ratio (NLR), and platelet-to-lymphocyte ratio (PLR) were unchanged. Meditation suggested anti-fatigue effects via improved emotion. Esketamine also decreased intraoperative remifentanil, accelerated bowel recovery, and caused no adverse reactions [56].

Pain Reduction

OFA achieves effective postoperative pain management through the use of non-opioid drugs and regional block techniques [53]. In orthopedic surgeries, such as joint replacement surgeries, OFA substantially decreases the amount of opioids needed after surgery, reduces pain levels, and shortens hospital stays, all while minimizing opioid-related side effects. The reported adverse effects were negligible, with no clinical complications, which underscores OFA's effectiveness in managing pain post-operation [57,58]. However, it should be reported that the pain reduction effect of OFA is debated in the literature, as some recent meta-analyses indicate that the benefits regarding the occurrence of postoperative pain, nausea, and vomiting are limited [59-61]. Some researchers have indicated that dexmedetomidine could lead to undesirable effects such as a higher chance of low blood pressure and slow heart rate during an operation, as well as extended sedation post-surgery [62]. 

**Figure 2:**
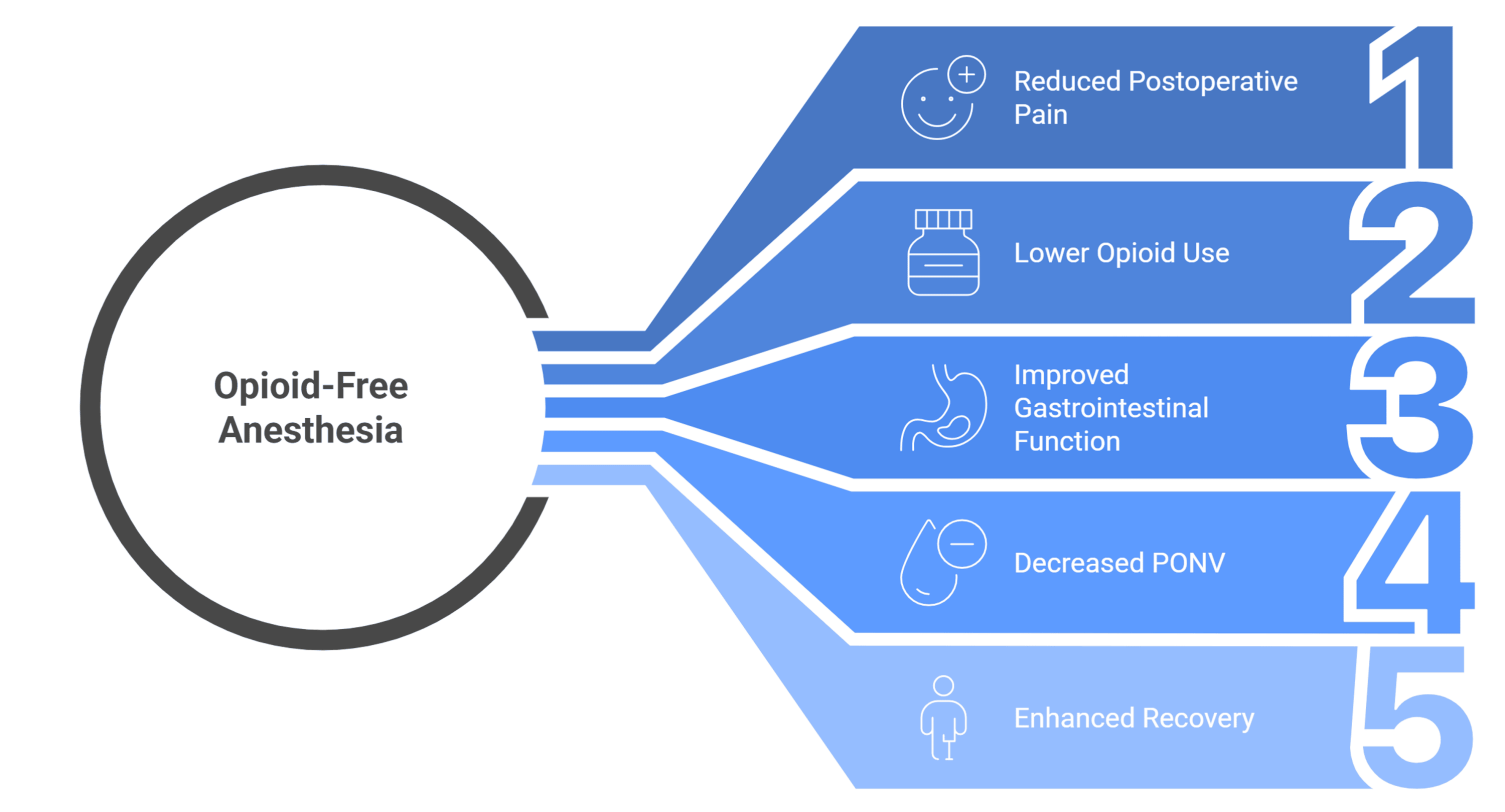
Exploring the benefits of opioid-free anaesthesia A figure created by the article authors showing the benefits of opioid-free anesthesia based on references [48-58].

Meta-analyses by Olausson et al. and Salomé et al. arrived at divergent conclusions. Olausson et al. reported that OFA lowers a range of postoperative adverse events without increasing intra- or postoperative complications, whereas Salomé et al. found no clinically meaningful pain benefit, noted reduced PONV, but observed more side effects when dexmedetomidine was part of the opioid-free protocol (Figure 2) [4,53].

Limitations and future directions

Evidence for OFA remains equivocal: existing studies are small and heterogeneous, producing fragile and inconsistent signals. Recent meta-analyses show limited reductions in postoperative pain, nausea, and vomiting, and their own methodological limitations have been reported [7,59-61,63]. As a result, high-quality data are insufficient to determine net benefit versus harm. Moreover, commonly used alpha-2 agonists, particularly dexmedetomidine, are linked to intraoperative hypotension and bradycardia, as well as prolonged postoperative sedation. Notably, the largest recent randomized trial of OFA reported several cases of severe bradycardia in the OFA arm, reinforcing the need for caution with this approach [62,64].

A retrospective study on the impact of opioid-free anesthesia (OFA) during the postoperative phase of cardiac surgery indicated potential adverse effects. These included a higher incidence of negative hemodynamic events or issues resulting from toxic plasma levels. The observed increase in norepinephrine and antihypertensive use might be linked to factors like higher isoproterenol dosages, the half-life of urapidil/nicardipine, and lidocaine's vasoactive properties [15].

Regional nerve blocks carry risks such as systemic toxicity from local anesthetics and bleeding. Non-opioid options like NSAIDs have an analgesic ceiling and may impair platelet function, disrupt gastrointestinal function, increase bleeding risk, and affect renal function. By contrast, acetaminophen does not inhibit platelet aggregation, slow gut motility, or alter cardiovascular activity and is not linked to NSAID-related bleeding, but its pain relief is modest, and it lacks anti-inflammatory effects [7].

The National Institute for Health and Care Excellence (NICE) identifies health-related quality of life, pain reduction, the quantity of additional medications used, and adverse treatment-related events as crucial outcomes for decision-making. Opioid-free anesthesia (OFA) decreases the amount of opioid medications and can improve certain outcomes, such as nausea and vomiting. While some aspects of quality of life, like recovery, are enhanced, these benefits are not universal. Considering that many of these objectives can be met through opioid-sparing approaches (prioritizing the avoidance of high opioid doses rather than complete elimination), a patient-centered strategy is essential. Given current guidelines and limited training, a more practical goal might be to prioritize opioid-sparing strategies and focus on reducing opioid use in anesthesia, rather than exclusively concentrating on OFA. It is also important to note that OFA does not preclude the administration of opioids in the postoperative phase [54].

Future research should refine OFA by testing lower, carefully titrated dexmedetomidine dosing and protocols that omit routine lidocaine, as these strategies may yield a more favorable risk-benefit balance. Studies should also examine whether extending agents like lidocaine, dexmedetomidine, or ketamine into the early postoperative period improves analgesia without introducing unacceptable adverse effects. Given the current uncertainty, well-designed trials are needed to determine OFA’s true efficacy and safety profile [3]. For OFA to be implemented effectively, healthcare professionals must get in-depth training. The pharmacological characteristics, modes of action, administration, and possible side effects of non-opioid drugs should all be covered in this course through methodical theoretical education. Equally important is practical training, which focuses on mastering methods like nerve blocks and regional anesthesia. The safe and efficient clinical use of OFA will be ensured by healthcare personnel's ability to continuously improve their abilities through frequent training programs, workshops, and academic exchanges.

## Conclusions

OFA provides a viable multimodal pathway that consistently reduces PONV, speeds gastrointestinal recovery, and can enhance overall recovery. These advantages align well with ERAS protocols and are particularly relevant in bariatric and select pediatric surgeries, where avoiding opioid-induced respiratory depression and oversedation is paramount. Importantly, OFA does not preclude the use of postoperative rescue opioids when clinically indicated; it should be viewed as a flexible component of patient-centered, multimodal care rather than an absolutist doctrine.

At the same time, current evidence remains heterogeneous and methodologically limited, with several meta-analyses showing modest or non-clinically meaningful differences in pain outcomes and signaling potential safety concerns, especially bradycardia and hypotension when α2-agonists are used. We therefore recommend a pragmatic emphasis on opioid-sparing strategies as the default, reserving strict OFA for appropriate patients and teams experienced with its protocols. Future work should focus on adequately powered, standardized RCTs that refine dosing (e.g., lower dexmedetomidine), clarify which surgical populations benefit most, extend evaluation into the early postoperative period, and couple implementation with structured training and outcome auditing to balance efficacy with safety.

## References

[1] Costa J, Benvenuto LJ, Sonett JR (2017). Long-term outcomes and management of lung transplant recipients. Best Pract Res Clin Anaesthesiol.

[2] Joshi GP (2023). Rational multimodal analgesia for perioperative pain management. Curr Pain Headache Rep.

[3] Salomé A, Harkouk H, Fletcher D, Martinez V (2021). Opioid-free anesthesia benefit-risk balance: a systematic review and meta-analysis of randomized controlled trials. J Clin Med.

[4] Lavand'homme P, Steyaert A (2017). Opioid-free anesthesia opioid side effects: tolerance and hyperalgesia. Best Pract Res Clin Anaesthesiol.

[5] de Boer HD, Detriche O, Forget P (2017). Opioid-related side effects: postoperative ileus, urinary retention, nausea and vomiting, and shivering. A review of the literature. Best Pract Res Clin Anaesthesiol.

[6] Paul AK, Smith CM, Rahmatullah M (2021). Opioid analgesia and opioid-induced adverse effects: a review. Pharmaceuticals (Basel).

[7] Beloeil H (2019). Opioid-free anesthesia. Best Pract Res Clin Anaesthesiol.

[8] Feng CD, Xu Y, Chen S (2024). Opioid-free anaesthesia reduces postoperative nausea and vomiting after thoracoscopic lung resection: a randomised controlled trial. Br J Anaesth.

[9] Liu Z, Bi C, Li X, Song R (2023). The efficacy and safety of opioid-free anesthesia combined with ultrasound-guided intermediate cervical plexus block vs. opioid-based anesthesia in thyroid surgery-a randomized controlled trial. J Anesth.

[10] Zhou F, Cui Y, Cao L (2023). The effect of opioid-free anaesthesia on the quality of recovery after endoscopic sinus surgery: a multicentre randomised controlled trial. Eur J Anaesthesiol.

[11] Cha NH, Hu Y, Zhu GH, Long X, Jiang JJ, Gong Y (2023). Opioid-free anesthesia with lidocaine for improved postoperative recovery in hysteroscopy: a randomized controlled trial. BMC Anesthesiol.

[12] Zhang Q, Wu Y, An H, Feng Y (2023). Postoperative recovery after breast cancer surgery: a randomised controlled trial of opioid-based versus opioid-free anaesthesia with thoracic paravertebral block. Eur J Anaesthesiol.

[13] Wang D, Sun Y, Zhu YJ, Shan XS, Liu H, Ji FH, Peng K (2024). Comparison of opioid-free and opioid-inclusive propofol anaesthesia for thyroid and parathyroid surgery: a randomised controlled trial. Anaesthesia.

[14] Zhong S, Sun Q, Wen J (2024). Dexmedetomidine attenuates inflammatory response and chronic pain following video-assisted thoracoscopic surgery for lung cancer. Surgery.

[15] Guinot PG, Spitz A, Berthoud V (2019). Effect of opioid-free anaesthesia on post-operative period in cardiac surgery: a retrospective matched case-control study. BMC Anesthesiol.

[16] Hall JE, Uhrich TD, Barney JA, Arain SR, Ebert TJ (2000). Sedative, amnestic, and analgesic properties of small-dose dexmedetomidine infusions. Anesth Analg.

[17] Scheinin B, Lindgren L, Randell T, Scheinin H, Scheinin M (1992). Dexmedetomidine attenuates sympathoadrenal responses to tracheal intubation and reduces the need for thiopentone and peroperative fentanyl. Br J Anaesth.

[18] Goettel N, Bharadwaj S, Venkatraghavan L, Mehta J, Bernstein M, Manninen PH (2016). Dexmedetomidine vs propofol-remifentanil conscious sedation for awake craniotomy: a prospective randomized controlled trial. Br J Anaesth.

[19] Panchgar V, Shetti AN, Sunitha HB, Dhulkhed VK, Nadkarni AV (2017). The effectiveness of intravenous dexmedetomidine on perioperative hemodynamics, analgesic requirement, and side-effects profile in patients undergoing laparoscopic surgery under general anesthesia. Anesth Essays Res.

[20] Zeng Q, Li J, Liu Y, Zhang Y, Su H, Tu F (2025). Effect of intravenous dexmedetomidine premedication on sufentanil median effective concentration during tracheal intubation in obese patients: a randomized controlled study. Drug Des Devel Ther.

[21] Hu J, Zhu M, Gao Z (2021). Dexmedetomidine for prevention of postoperative delirium in older adults undergoing oesophagectomy with total intravenous anaesthesia: a double-blind, randomised clinical trial. Eur J Anaesthesiol.

[22] Schnabel A, Meyer-Frießem CH, Reichl SU, Zahn PK, Pogatzki-Zahn EM (2013). Is intraoperative dexmedetomidine a new option for postoperative pain treatment? A meta-analysis of randomized controlled trials. Pain.

[23] Lu Y, Fang PP, Yu YQ (2021). Effect of intraoperative dexmedetomidine on recovery of gastrointestinal function after abdominal surgery in older adults: a randomized clinical trial. JAMA Netw Open.

[24] Yang J, Zhao M, Zhang XR (2022). Ropivacaine with dexmedetomidine or dexamethasone in a thoracic paravertebral nerve block combined with an erector spinae plane block for thoracoscopic lobectomy analgesia: a randomized controlled trial. Drug Des Devel Ther.

[25] Marhofer D, Kettner SC, Marhofer P, Pils S, Weber M, Zeitlinger M (2013). Dexmedetomidine as an adjuvant to ropivacaine prolongs peripheral nerve block: a volunteer study. Br J Anaesth.

[26] Blaudszun G, Lysakowski C, Elia N, Tramèr MR (2012). Effect of perioperative systemic α2 agonists on postoperative morphine consumption and pain intensity: systematic review and meta-analysis of randomized controlled trials. Anesthesiology.

[27] Hermanns H, Hollmann MW, Stevens MF, Lirk P, Brandenburger T, Piegeler T, Werdehausen R (2019). Molecular mechanisms of action of systemic lidocaine in acute and chronic pain: a narrative review. Br J Anaesth.

[28] Kaba A, Laurent SR, Detroz BJ, Sessler DI, Durieux ME, Lamy ML, Joris JL (2007). Intravenous lidocaine infusion facilitates acute rehabilitation after laparoscopic colectomy. Anesthesiology.

[29] Wang S, Li Y, Liang C, Han X, Wang J, Miao C (2023). Opioid-free anesthesia reduces the severity of acute postoperative motion-induced pain and patient-controlled epidural analgesia-related adverse events in lung surgery: randomized clinical trial. Front Med (Lausanne).

[30] Mion G, Himmelseher S (2024). Esketamine: less drowsiness, more analgesia. Anesth Analg.

[31] Song N, Yang Y, Zheng Z (2023). Effect of esketamine added to propofol sedation on desaturation and hypotension in bidirectional endoscopy: a randomized clinical trial. JAMA Netw Open.

[32] Luo LL, Xiao R, Zhang JP, Xi WF, Xu GH, Yuan H (2025). Opioid-free anesthesia with esketamine combined with iliac fascia block in elderly patients undergoing hip surgery. Drug Des Devel Ther.

[33] Accurso G, Rampulla D, Cusenza M (2025). A blended opioid-free anesthesia protocol and regional parietal blocks in laparoscopic abdominal surgery- a randomized controlled trial. Sci Rep.

[34] Rawal N (2021). Epidural analgesia for postoperative pain: improving outcomes or adding risks?. Best Pract Res Clin Anaesthesiol.

[35] Dam M, Hansen CK, Poulsen TD (2019). Transmuscular quadratus lumborum block for percutaneous nephrolithotomy reduces opioid consumption and speeds ambulation and discharge from hospital: a single centre randomised controlled trial. Br J Anaesth.

[36] Pei L, Zhou Y, Tan G (2015). Ultrasound-assisted thoracic paravertebral block reduces intraoperative opioid requirement and improves analgesia after breast cancer surgery: a randomized, controlled, single-center trial. PLoS One.

[37] Oksar M, Koyuncu O, Turhanoglu S, Temiz M, Oran MC (2016). Transversus abdominis plane block as a component of multimodal analgesia for laparoscopic cholecystectomy. J Clin Anesth.

[38] Ramaswamy S, Wilson JA, Colvin JA (2013). Non-opioid-based adjuvant analgesia in perioperative care. Continuing Education in Anaesthesia, Critical Care and Pain.

[39] Nair A, Rangaiah M, Borkar N (2023). Efficacy and safety of oral tizanidine premedication as pre-emptive analgesia in adult patients undergoing elective surgeries- a systematic review. Saudi J Anaesth.

[40] Baradwan S, Alshahrani MS, Alkhamis WH (2022). Preoperative duloxetine on postoperative pain after laparoscopic gynecological surgeries: a systematic review and meta-analysis of randomized controlled trials. J Gynecol Obstet Hum Reprod.

[41] Dey S, Maurya I, Lohiya A, Arora P, Abdulkader RS, Chanu SM (2023). Efficacy of flupirtine for postoperative pain: a systematic review and meta-analysis. Indian J Anaesth.

[42] Song B, Li W, Wan L, Zhang L (2025). Effect of opioid-free versus opioid anesthesia on the quality of postoperative recovery in patients receiving laparoscopic sleeve gastrectomy. Obes Surg.

[43] Zhou X, Feng W, Wang X, Niu Z, Wang P, Yuan L, Wang P (2024). The effect of opioid-free anesthesia with transversus abdominis plane block on patients undergoing laparoscopic sleeve gastrectomy: randomized controlled study. J Pain Res.

[44] Tsui BC, Pan S, Smith L, Lin C, Balakrishnan K (2021). Opioid-free tonsillectomy with and without adenoidectomy: the role of regional anesthesia in the “new era”. Anesth Analg.

[45] Franz AM, Martin LD, Liston DE, Latham GJ, Richards MJ, Low DK (2021). In pursuit of an opioid-free pediatric ambulatory surgery center: a quality improvement initiative. Anesth Analg.

[46] Pestieau SR, Quezado ZM, Johnson YJ (2011). High-dose dexmedetomidine increases the opioid-free interval and decreases opioid requirement after tonsillectomy in children. Can J Anaesth.

[47] Hardt K, Wappler F (2023). Anesthesia for morbidly obese patients. Dtsch Arztebl Int.

[48] Gupta K, Nagappa M, Prasad A, Abrahamyan L, Wong J, Weingarten TN, Chung F (2018). Risk factors for opioid-induced respiratory depression in surgical patients: a systematic review and meta-analyses. BMJ Open.

[49] Katz JD (2016). The aging anesthesiologist. Curr Opin Anaesthesiol.

[50] Drummond GB, Bates A, Mann J, Arvind DK (2013). Characterization of breathing patterns during patient-controlled opioid analgesia. Br J Anaesth.

[51] Stenberg E, Dos Reis Falcão LF, O'Kane M (2022). Guidelines for perioperative care in bariatric surgery: Enhanced Recovery After Surgery (ERAS) Society recommendations: a 2021 update. World J Surg.

[52] Hao C, Xu H, Du J, Zhang T, Zhang X, Zhao Z, Luan H (2023). Impact of opioid-free anesthesia on postoperative quality of recovery in patients after laparoscopic cholecystectomy—a randomized controlled trial. Drug Des Devel Ther.

[53] Olausson A, Svensson CJ, Andréll P, Jildenstål P, Thörn SE, Wolf A (2022). Total opioid-free general anaesthesia can improve postoperative outcomes after surgery, without evidence of adverse effects on patient safety and pain management: a systematic review and meta-analysis. Acta Anaesthesiol Scand.

[54] Forget P, Van de Velde M, Pogatzki-Zahn E (2023). Opioid-free anaesthesia: should we all adopt it? An overview of current evidence. Eur J Anaesthesiol.

[55] da Silveira CA, Rasador AC, Medeiros HJ, Slawka E, Gesteira L, Pereira LC, Amaral S (2024). Opioid-free anesthesia for minimally invasive abdominal surgery: a systematic review, meta-analysis, and trial sequential analysis. Can J Anaesth.

[56] Sun L, Zhao Y, Li Y (2023). Effect of continuous subanesthetic esketamine infusion on postoperative fatigue in patients undergoing laparoscopic radical resection for colorectal cancer: a randomized controlled study. Am J Cancer Res.

[57] Xu Y, Zhong M, Li S (2025). Opioid-free anesthesia in enhanced recovery after surgery for gastrointestinal surgery: current status, challenges, and prospects. Front Pharmacol.

[58] Urvoy B, Aveline C, Belot N, Catier C, Beloeil H (2021). Opioid-free anaesthesia for anterior total hip replacement under general anaesthesia: the Observational Prospective Study of Opiate-free Anesthesia for Anterior Total Hip Replacement trial. Br J Anaesth.

[59] Frauenknecht J, Kirkham KR, Jacot-Guillarmod A, Albrecht E (2019). Analgesic impact of intra-operative opioids vs. opioid-free anaesthesia: a systematic review and meta-analysis. Anaesthesia.

[60] Grape S, Kirkham KR, Frauenknecht J, Albrecht E (2019). Intra-operative analgesia with remifentanil vs. dexmedetomidine: a systematic review and meta-analysis with trial sequential analysis. Anaesthesia.

[61] Albrecht E, Grape S, Frauenknecht J, Kilchoer L, Kirkham KR (2020). Low- versus high-dose intraoperative opioids: a systematic review with meta-analyses and trial sequential analyses. Acta Anaesthesiol Scand.

[62] Demiri M, Antunes T, Fletcher D, Martinez V (2019). Perioperative adverse events attributed to α2-adrenoceptor agonists in patients not at risk of cardiovascular events: systematic review and meta-analysis. Br J Anaesth.

[63] Beloeil H, Garot M, Martinez V (2019). Comments on Albrecht et al. reviews. Anaesthesia.

[64] Beloeil H, Laviolle B, Menard C (2018). POFA trial study protocol: a multicentre, double-blind, randomised, controlled clinical trial comparing opioid-free versus opioid anaesthesia on postoperative opioid-related adverse events after major or intermediate non-cardiac surgery. BMJ Open.

